# Synthetic and immunological studies on the OCT4 immunodominant motif antigen-based anti-cancer vaccine

**DOI:** 10.20892/j.issn.2095-3941.2019.0224

**Published:** 2020-02-15

**Authors:** Tingting Chen, Kan Liu, Jiangyao Xu, Tianying Zhan, Maixian Liu, Li Li, Zhiwen Yang, Shuping Yuan, Wenyi Zou, Guimiao Lin, Dennis A. Carson, Christina C. N. Wu, Xiaomei Wang

**Affiliations:** ^1^Department of Physiology, School of Basic Medical Sciences, Shenzhen University Health Sciences Center, Shenzhen University, Shenzhen 518060, China; ^2^Carson Lab, Moores Cancer Center, UCSD, La Jolla 92093, CA, USA

**Keywords:** Cancer prevention, cancer immunology, OCT4, TLR9 agonist

## Abstract

**Objective:** Cancer stem cell is one of the important causes of tumorigenesis as well as a drug target in the treatment of malignant tumor. However, at present, there is no immune vaccine targeting these cells. Octamer-binding transcription factor 4 (OCT4), a marker of embryonic stem cells and germ cells, often highly expresses in the early stages of tumorigenesis and is therefore a good candidate for cancer vaccine development.

**Methods:** To identify the optimal carrier and adjuvant combination, we chemically synthesized and linked three different OCT4 epitope antigens to a carrier protein, keyhole limpet hemocyanin (KLH), combined with Toll-like receptor 9 agonist (TLR9).

**Results:** Immunization with OCT4-3 + TLR9 produced the strongest immune response in mice. In prevention assays, significant tumor growth inhibition was achieved in BABL/c mice treated with OCT4-3 + TLR9 (*P* < 0.01). Importantly, the results showed that cytotoxic T lymphocyte activity and the inhibition of tumor growth were enhanced in mice immunized with OCT4-3 combined with TLR9. Meanwhile, multiple cytokines [such as interferon (IFN)-γ (*P* < 0.05), interleukin (IL)-12 (*P* < 0.05), IL-2 (*P* < 0.01), and IL-6 (*P* < 0.05)] promoting cellular immune responses were shown to be greatly enhanced in mice immunized with OCT4-3 + TLR9. Moreover, we considered safety considerations in terms of the composition of the vaccines to help facilitate the development of effective next-generation vaccines.

**Conclusions:** Collectively, these experiments demonstrated that combination therapy with TLR9 agonist induced a tumor-specific adaptive immune response, leading to the suppression of primary tumor growth in testis embryonic carcinoma.

## Introduction

Cancer is a major global public health issue. The global burden of cancer worldwide is continuing to increase, with an estimated 18.1 million new cancer cases and 9.6 million cancer deaths in 2018^1^. At present, in addition to traditional surgery, radiotherapy, and chemotherapy, new treatments such as targeted drugs^2^, checkpoint blockade [PD-1/PD-L1 antibody^3^, cytotoxic T lymphocyte-4 (CTLA)-4 antibody^4^] and adoptive immunotherapy [chimeric antigen receptor T-cell (CAR-T)/TCR-T^5^] have also changed the life expectancy of cancer patients to some extent. Cancer immunoprevention is a promising new field^6^. Over the past 20 years, prophylactic vaccines have been used to prevent cancer, showing efficiency by promoting tumor-specific immune responses with low level toxicity^7^. To design an effective anti-cancer gene vaccine, vaccines based on tumor-specific antigens (TSA) or tumor-associated antigens (TAA) have been tested in patients with advanced or recurrent cancers in combination with, or following, standard therapies^8^. Immunoprevention can benefit from advances in cancer immunotherapy, which aims to prevent cancers by using vaccines against carcinogenic viruses and tumor antigens, and antibodies against immune molecules and immunomodulators^9^.

Cancer stem cells (CSCs) are one of the important causes of cancer initiation, dissemination, and recurrence. Only a small number of cells in solid tumors are CSCs, but they have strong self-renewal abilities^10^. Moreover, because of the expression of normal stem cell proteins, such as OCT4 and Nanog, the immune system cannot recognize them effectively, which results in the strong immune evasion ability of CSCs and the high clinical recurrence rate of patients. Therefore, CSCs are an important drug target in the treatment of malignant tumors^11^, and are an important factor in completely curing tumors. However, at present, there is a lack of immune vaccines targeting these cells.

Octamer-binding transcription factor 4 (OCT4), also known as POU5F1, is one of the key transcription factors involved in the regulation of self-renewal and totipotency of embryonic stem cells in the POU family. As a marker of embryonic stem cells and germ cells, OCT4 is downregulated in differentiated cells, suggesting that OCT4 may be involved in regulating cell differentiation^12,13^. Recent studies have shown that OCT4 is also expressed in a variety of CSCs outside the reproductive system^14^, especially in the early stages of tumorigenesis^15,16^. Testicular embryonal carcinoma (EC) is a malignant teratoma stem cell. The expression of OCT4 in the mouse testicular embryonic cancer cell line F9 is significantly higher than in normal mice. It has been confirmed that OCT4 is a marker of early metastasis of testicular cancer^15^. Our group first coupled OCT4 full protein with Toll-like receptor 7 (TLR7) agonist, and a significant immune response was obtained in mice. This vaccine also had a satisfactory inhibitory effect on the growth of OCT4-positive testicular cancer cells, and it was confirmed that targeted immune killing was directly related to the role of cytotoxic T lymphocytes^17^. However, the body’s immune tolerance and weak immunogenicity of tumor antigens limited their use as effective antigen epitopes for preventive and therapeutic vaccines. To address this problem, we employed the immune dominant region of OCT4. We designed and synthesized its linear fragments and coupled the antigen epitope peptide fragments with keyhole limpet hemocyanin (KLH), in combination with Toll-like receptor 9 (TLR9) agonist. Notably, significant tumor inhibition was achieved in OCT4-3 + TLR9-treated BABL/c mice, prophylactically. Our findings provided evidence for the first time that OCT4-3 + TLR9 induced significantly stronger immune responses and suppressed tumor growth in BABL/c mice without any significant systemic toxicity.

## Material and methods

### Epitope prediction

The protein sequences of OCT4 were obtained from the National Center for Biotechnology Information (NCBI; http://www.ncbi.nlm.nih.gov). Using the epitope prediction program IEDB (http://www.iedb.org/), the sequences of OCT4 were analyzed and three peptides with high affinity for MHC class I were identified. The peptide sequences were OCT4-1: DNNENLQEICKAETLVQARKRKRTSIE; OCT4-2: ENRVRGNLENLFLQCPKPTLQQISHIAQQLGLE; and OCT4-3: EAAGSPFSGGPVSFPLAPGPHFGTPGYGSPHF.

### Experimental animals and cell lines

Male BALB/c mice (4–6-weeks-old) were purchased from the Medical Laboratory Animal Center (Guangzhou, Guangdong Province, China). All animal treatments and experimental protocols for this study were approved by the Laboratory Animals Center and the Experimental Animal Ethics Committee, School of Medicine, Shenzhen University (Permit No. AEWC-2019003). All mice were housed under a 12-h light/dark cycle, at 23 ± 1°C, with 39%–43% relative humidity. Water and food were provided *ad libitum*. OCT4^+^ F9 cells (mouse teratocarcinoma cells) were maintained in Dulbecco’s Modified Eagle’s Medium (DMEM) with 10% fetal bovine serum (FBS) and 100 U/mL penicillin-streptomycin, and all of these reagents were obtained from Hyclone Laboratories, Inc. (South Logan, UT, USA).

### Vaccine preparation and administration

Three OCT4 peptides were synthetized at ChinaPeptides Co., Ltd. (Shanghai, China). To elicit antibody production and immune responses against the hapten, KLH (H7017; Sigma-Aldrich, St. Louis, MO, USA) was chosen and 1-ethyl-3-(-3-dimethylaminopropyl) carbodiimide hydrochloride (EDC) (#22980; Thermo Fisher Scientific, Waltham, MA, USA) and N-hydroxysuccinimide (NHS) (#24500; Thermo Fisher Scientific, Waltham, MA, USA) were used to activate carboxylic acid as an active ester. The molar ratios of KLH, polypeptide, EDC, and NHS were 1:10:100:100, respectively, and this mixture was incubated at 4°C overnight. To remove excess KLH and EDC/NHS catalyst, the reaction mixture was washed with phosphate-buffered saline (PBS) three times on a 10,000 MWCO Microcon filtration device (Millipore, Billerica, MA, USA). The adjuvant, Toll-like receptor (TLR) 9 agonist, and Quick Antibody-Mouse 5W, was supplied by Biodragon-Immunotech Inc. (KX0210041; Beijing, China) and was mixed with different OCT4 peptides at a 1:1 volume.

### Tumor protection in BALB/c mice

Male BALB/c mice (6–10 per group) were vaccinated with 50 µg of different peptides *via* intraperitoneal (i.p.) injection on days 0, 14, and 21. To assess the immune memory response, a booster dose was given at 28 days. Five days after the final vaccination, the mice were challenged subcutaneously (s.c.) with 5 × 10^5^ OCT4^+^ F9 cells (a total of 200 µL of cell mixture in 50% Matrigel (#354234; Corning, Corning, NY, USA) into the lower flank of the right back and the tumor size was measured periodically with calipers. Starting from day 0 after cell inoculation, the tumor volume was measured every 2 days. Tumor size was calculated using the formula: 0.5 × length × width^2^ (cm^3^)^18^. Mice were euthanized when the tumor size was > 1.5 cm^3^. Mouse blood samples were collected from retro-orbital tissues at the indicated time points.

### Cytotoxicity assay *in vivo*

Splenocytes of vaccinated mice were harvested for evaluating cellular immune responses 4–5 days after the final vaccination. The cytotoxicity activity of vaccinated mice was analyzed using a CytoTox96 Non-Radioactive Cytotoxicity Assay Kit (G1780; Promega, Madison, WI, USA). Briefly, splenocytes from the vaccinated mice were mixed at an effector to target ratio of 12.5:1 (E:T = 12.5:1) with 5 × 10^3^ F9 cells loaded with the OCT4-3 peptide for 24 h. CTL activity was evaluated by analyzing the released lactate dehydrogenase. The cytolysis rate (%) was calculated based on the equation: cytotoxicity (%) = (experimental release − spontaneous release)/(maximum release − spontaneous release) × 100.

### Enzyme-linked immunosorbent assay (ELISA)

Sera samples were collected at 3–5 days after four doses of vaccine had been administered. An ELISA for multiple cytokines in peripheral blood was performed using a Ready-SET-GO! ELISA kit according to the instructions provided by the manufacturer (eBioscience, Thermo Fisher Scientific).

### Hematoxylin and eosin (H&E) staining and immunofluorescence staining

Tumor tissues were harvested and fixed in 10% buffered formalin, dehydrated, cleared and embedded in paraffin according to standard procedures^19^. They were then sectioned at 3 µm, mounted onto polylysine-coated slides, and the sections were stained with H&E. Histopathological morphology was checked using a microscope. For immunofluorescence assays, the sections were deparaffinized in xylene and rehydrated in graded ethanol. Antigen retrieval was performed in citrate buffer (pH 6.0) by boiling for 30 min in a microwave and cooling to room temperature. Then, the sections were blocked by 3% bovine serum albumin (BSA) in PBS for 1 h and 0.3% hydrogen peroxide (H2O2) solution in PBS for 10 min. The sections were then stained with antibodies against mouse CD3 (1:500, ab16669; Abcam, Cambridge, UK) at 4°C overnight. After washing, the sections were incubated with secondary antibody, AlexaFluor 488 (goat anti-rabbit, 1:500, ab16669; Abcam) for 1 h, and were then washed and mounted in Fluoroshield with DAPI (F6057; Sigma-Aldrich). Images were acquired using a Nikon Eclipse E400 microscope (Nikon, Tokyo, Japan) with an Olympus DP73 camera (Olympus, Tokyo, Japan) and cellSence Entry software (Olympus).

### Measurement of immunoglobulin G (IgG)

Anti-OCT4 IgG was measured by an ELISA as previously reported^20^. Briefly, each 96-well ELISA plate (Costar, Corning, NY, USA) was coated with 2 µg of synthetic OCT4 peptide at 4°C overnight. Plates were then washed with PBST (0.01 M PBS containing 0.05% Tween 20, pH 7.4) three times. Serum samples were detected at a 1:500 dilution for 2 h. Alkaline phosphatase-conjugated detection antibody for total IgG (Sigma-Aldrich) was added and incubated for 1 h at room temperature. Then, p-NPP substrate (Millipore) and stop solution (50 µL of 3 M NaOH) were added to each well, and the optical density (OD, 405 nm) was measured by a spectrophotometer (BioTek, Winooski, VT, USA).

### Monitoring for adverse events

Laboratory monitoring for adverse events included conducting complete blood counts, measuring blood urea nitrogen, and performing liver function tests at baseline, before tumor inoculation. For the determination of mouse sperm morphology, sperms were extracted from the seminal vesicle, spread onto histological slides in 0.1 M sodium phosphate buffer, pH 7.2, fixed in a solution of 4% paraformaldehyde for 15–20 min, washed with tap water, and dried at room temperature. The slides were then stained with Giemsa (G4507; Sigma-Aldrich) for 15 min, washed with tap water, and dried at room temperature. The analysis and photo-documentation of the sperm were performed using a photomicroscope (Olympus BX-60).

### Statistical analysis

All statistical analyses were performed using GraphPad Prism software (La Jolla, CA, USA) and the unpaired *t*-test. Data are shown as the mean ± standard deviation (SD). For all statistical methods, values of *P* < 0.05 were considered significant, and those of *P* < 0.01 were considered highly significant.

## Results

### Epitope prediction and construction

**Figure 1:**
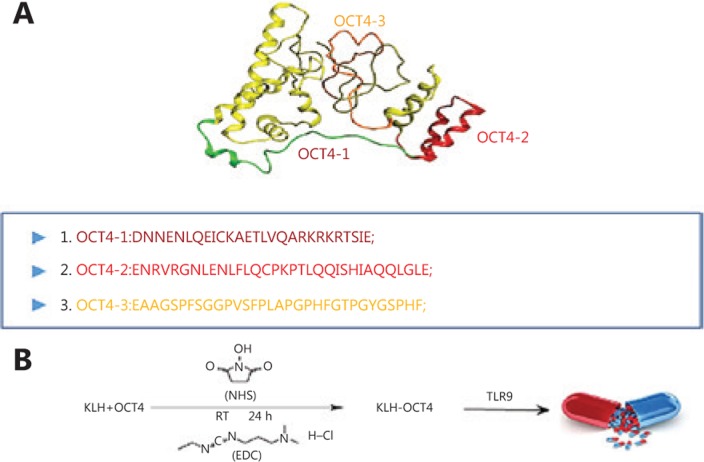
The ideograph of OCT4. (A) The three-dimensional (3D) reconstruction of OCT4. (B) Schematic of OCT4-1, OCT4-2, and OCT4-3.

The immunogenicity of peptides of each of the antigens, identified by epitope prediction programs, was tested by immunizing mice as described in the Materials and methods section. Three immunogenic peptides, OCT4-1, 2, and 3, were identified (**Figure 1A**). Using the I-TASSER website, a three-dimensional (3D) molecular model of the peptide was predicted, and the predicted 3D structure is shown in **Figure 1A**. KLH, used as a carrier protein due to its highly immunogenic properties and the large number of lysine residues available for modification, is suitable for the preparation of immunogens for injection. Haptens of the OCT4-1, 2, 3 peptides were coupled to KLH by EDC and NHS (**Figure 1B**).

### Anti-tumor effects of OCT4 vaccine

**Figure 2:**
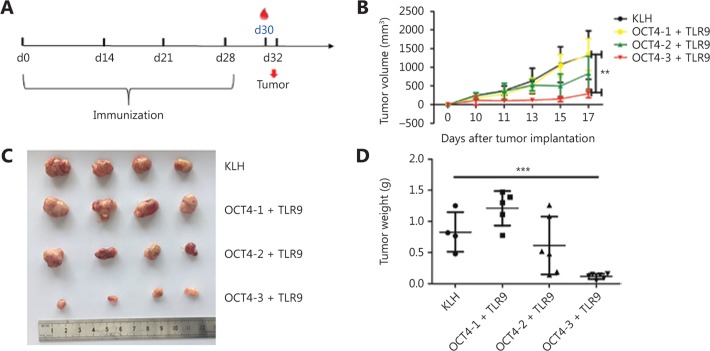
Anti-tumor effects of the OCT4 vaccine. (A) Schematic diagram of the dosing regimen for the prophylactic treatment of OCT4 in BALB/c mice. (B) Tumor volume (mm^3^) was assessed every other day. Tumor sizes are presented as (C) an image, and (D) tumor weight following euthanization and tumor isolation 17 days after treatment.

The anti-tumor effects of these three OCT4 vaccines were evaluated using a protection experiment. BALB/c mice were vaccinated with the combination vaccine using the immunization schedule outlined in **Figure 2A**, with KLH as a negative control. Tumor volumes were measured with a vernier caliper every 2 days after the tumors began to grow. The results showed that tumors grew slowly and were significantly decreased in the OCT4-3 + TLR9 vaccine group (**Figure 2B**). On day 17, all mice were euthanized by CO2 asphyxiation followed by cerveical dislocatoin, and tumors were collected.

The tumor weights were significantly decreased in the OCT4-3 + TLR9 vaccine group in the treatment experiment (**Figure 2C** and **2D**). Therefore, according to the tumor inhibition rate analysis described above, the combination of OCT4-3 and TLR9 vaccine was chosen for further study.

### OCT4-3 + TLR9 induces effective tumor suppression prophylactically

**Figure 3:**
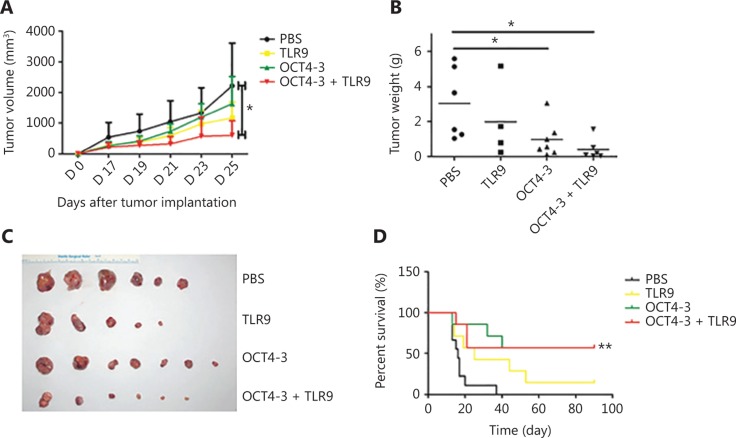
Prophylaxis studies on the antitumor effect of OCT4-3 + Toll-like receptor-9 (TLR9) in BABL/c mice. (A) Tumor growth curve of the BALB/c mice bearing an F9 cell xenograft. Compared with phosphate-buffered saline (PBS) and OCT4-3 + TLR9, significant reduction in tumor growth for OCT4-3 + TLR9 could be observed (^*^P < 0.05). (B) Scatter diagram of tumor weight. Measurement of subcutaneous tumor weight 25 days after inoculation in prophylactic settings (^*^P < 0.05). (C) Tumor images from each group following the removal of tumors from euthanized mice at the end of the study. (D) Overall survival curves of mice treated as mentioned with PBS, TLR9, OCT4-3, or OCT4-3 + TLR9. ^**^P < 0.01 (log-rank test) vs. PBS groups.

We further investigated the *in vivo* anti-tumor efficacy of OCT4-3 + TLR9. BABL/c mice were immunized i.p. with PBS, TLR9, OCT4-3, or OCT4-3 + TLR9, four times, followed by challenge with 5 × 10^5^ OCT4^+^ F9 cells s.c. After treatment for 25 days, the animals were humanely euthanized, and the tumors were separated. We found that OCT4-3 + TLR9 significantly inhibited tumor growth compared with the PBS control group (**Figure 3A**). The tumor weights (**Figure 3B**) and images of tumors (**Figure 3C**) of each group also indicated the significant advantages of OCT4-3 + TLR9 in suppressing tumor growth. According to the above data, we questioned whether this combined vaccine could also prolong the survival time of mice with F9 cancer. Thirty-seven days after tumor challenge, all mice in the PBS group had died, whereas at 90 days post-tumor implantation, 20% of mice exhibited no tumors in the OCT4 + TLR9 group (*P* < 0.01) (**Figure 3D**). This supported the hypothesis that treatment with the combined vaccine produced immune responses capable of restraining tumor growth.

### Enhanced immunogenicity of the OCT4-3 + TLR9 combined vaccine

**Figure 4:**
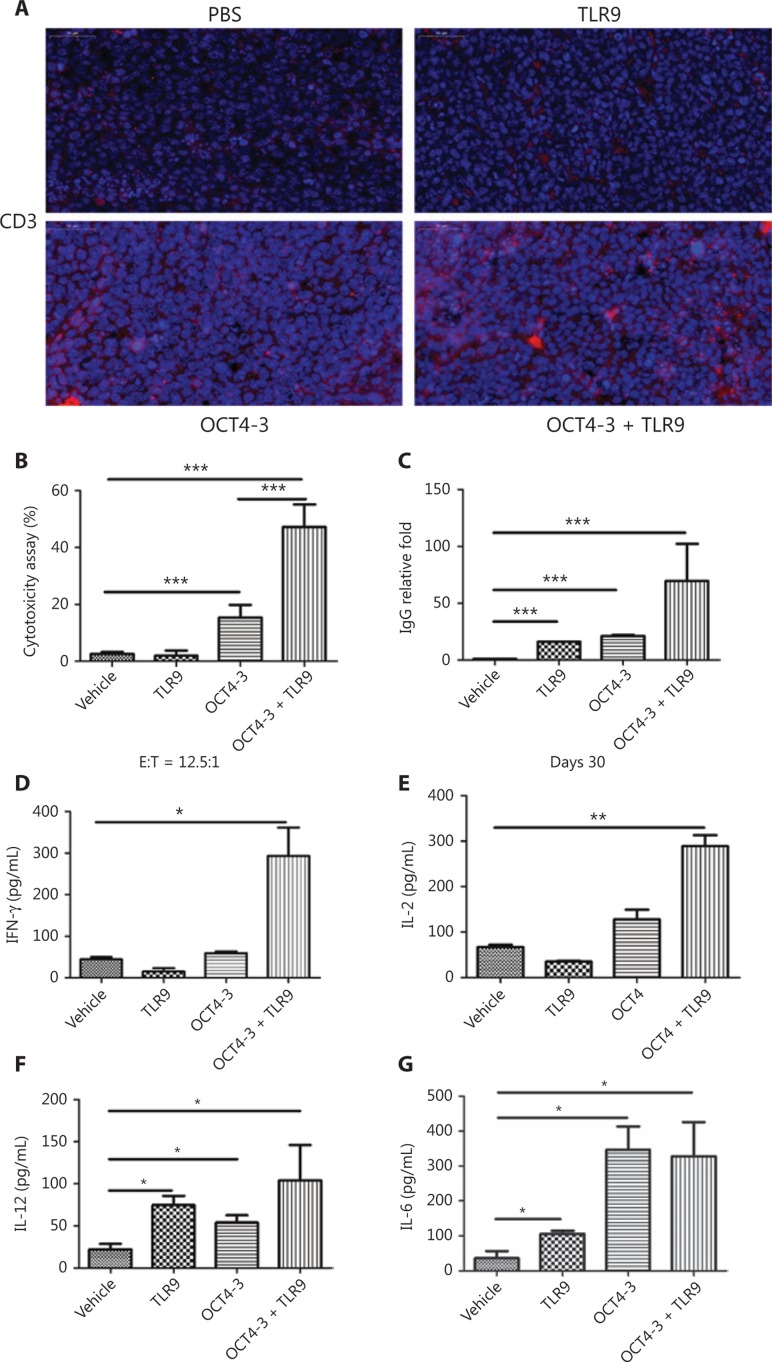
Enhancement of CTL activity and secretion of multiple cytokines. (A) Analysis of CD3+ T cells in tumor tissues from ectopic tumor-bearing mice treated with phosphate-buffered saline (PBS), Toll-like receptor-9 (TLR9), OCT4-3, and OCT4-3 + TLR9, respectively. (B) Cytotoxic T lymphocyte (CTL) assay. (C) Vaccine elicited anti-OCT4 immunoglobulin G (IgG) responses. Fold changes in serum. (D–G) Secretion of the cytokines: (D) interferon (IFN)-r, (E) interleukin (IL)-2, (F) IL-12, and (G) IL-6.

The immunogenicity of the OCT4-3 and TLR9 combined vaccine was studied. Notably, immunohistochemical staining of CD3^+^ cells in tumor tissues revealed a substantial number of CD3^+^ T cells in the tumor sections of OCT4-3 + TLR9-treated mice, suggesting that T cells were likely effector cells mediating tumor regression (**Figure 4A**). The results of the CTL assay using OCT4-3 peptide-labeled F9 target cells showed that specific CTL responses were induced by combined antigens compared with the PBS control (*P* < 0.05). Moreover, OCT4 + TLR9 induced a significantly higher specific CTL response than OCT4 alone (**Figure 4B**). The results demonstrated that the killing efficiency of the antigen-specific CD8^+^ cells was elicited by immunization with target antigen OCT4. The anti-OCT4 IgG response was the main measure of vaccine immunogenicity because elicitation of an IgG antibody requires activation not only of OCT4-specific B cells, but also of OCT4-specific helper T cells that promote anti-OCT4 antibody isotype switching from IgM to IgG. Remarkable increases in the IgG fold-changes were noted in the OCT4-3 + TLR9 vaccine group (**Figure 4C**). Furthermore, sera were collected and the levels of interferon (IFN)-γ, interleukin (IL)-12, IL-2, and IL-6 cytokines were detected. The ELISA results shown in **Figure 4D–G** indicated that the levels of secreted IFN-γ, IL-12, IL-2, and IL-6 were significantly higher in the OCT4-3 + TLR9 vaccine group compared with the PBS control group.

### Toxicity studies

Because OCT4 is one of the most important proteins necessary for maintaining the pluripotency of stem cells, it is unknown whether OCT4 polypeptide can produce irreversible side effects after immunization. Although it has been reported that OCT4 has no effect on normal adult cells, there is still no research regarding the effect on the human body of using stem cell markers as cancer polypeptide vaccines^21^.

**Figure 5:**
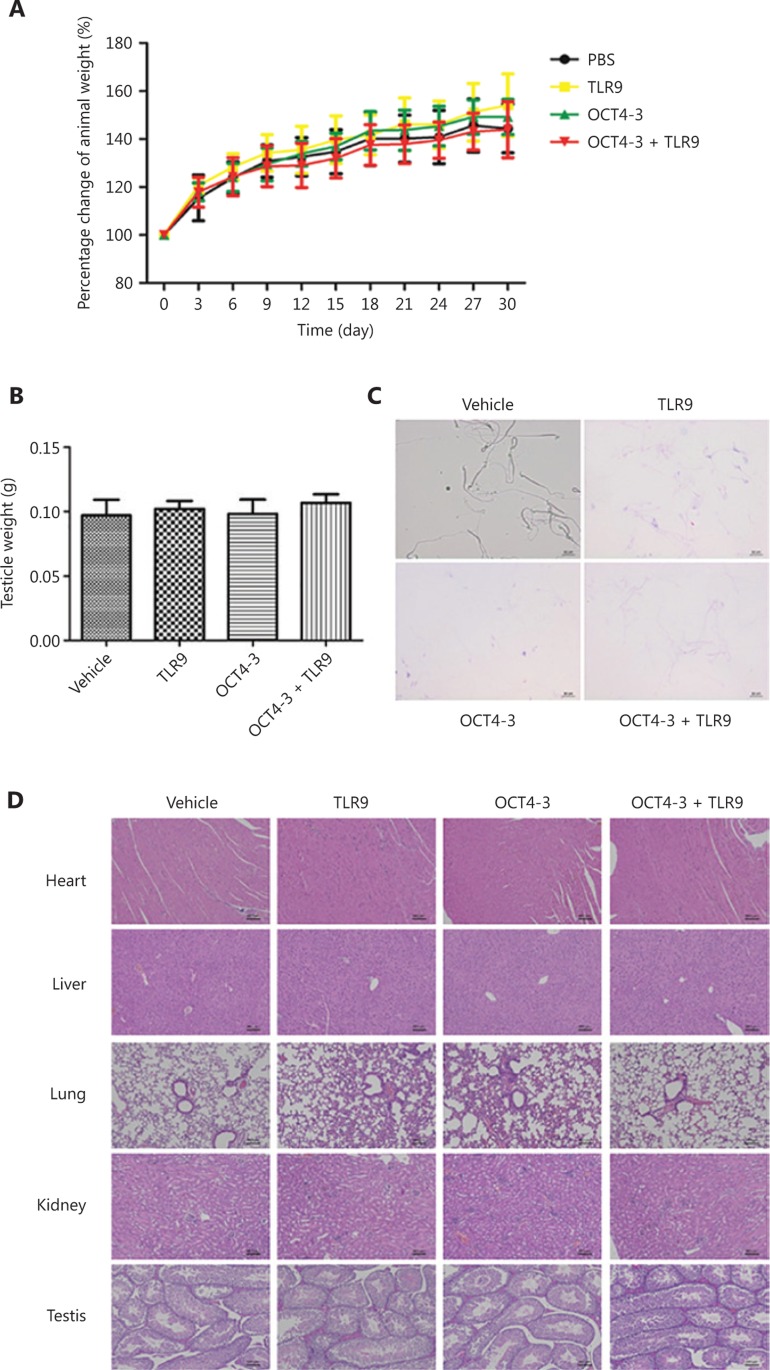
Toxicity study of the organs of treated mice. (A) Percentage change in the animal weight curve of BALB/c mice after immunization with vaccine. (B) The weight of unilateral testicles. (C) Mouse sperm under light microscopy (scale bar: 10 μm). (D) Morphologies of the heart, liver, lung, kidney and testis after hematoxylin and eosin (H&E) staining were compared to evaluate organ-specific toxicity. Scale bar, 50 μm.

The OCT4-3 vaccine was well tolerated in mice, and no significant adverse events were observed during or after vaccination. Treatment with OCT4-3 vaccine did not reduce the body weight of mice (**Figure 5A**). Testicles were the only organs that produced spermatogonia, and the unilateral testicles of mice were removed and weighed (**Figure 5B**). The results showed that there was no significant difference in weight among the groups (*P* > 0.05). The morphology of the sperm was also analyzed and photographed, and there was no difference among the groups (**Figure 5C**).

Important organs (such as the heart, liver, lung, kidney, and testis) were removed from the vaccinated mice for toxicity testing by H&E staining, and the results showed no noticeable morphological change among any of the four groups (**Figure 5D**). In addition to the histological observations, hematological and biochemical analyses were also performed to evaluate the biosafety of vaccines. The serum levels of alanine aminotransferase (ALT), aspartate aminotransferase (AST), alkaline phosphatase (ALP), blood urea nitrogen (BUN), albumin (ALB), glucose (GLU), total bilirubin levels (TBIL), creatinine (CREA), and total protein (TP) were measured as indicators of renal and liver function (**Table 1**). Whole blood was measured for changes in white blood cell (WBC) count, red blood cell (RBC) count, hemoglobin (HGB) count, red blood cell specific volume (HCT), mean corpuscular volume (MCV) and platelet (PLT) count (**Table 2**). No significant difference was found.

## Discussion

Immunoprevention of cancer through the use of cancer vaccines has the potential for noninvasive, nontoxic, and due to the specificity of the immune response and its long-term memory, prolonged protection. Among several common immunotherapy methods, the effectiveness of PD-1/PD-L1 inhibitors depends on tumor infiltrating lymphocytes, which can recognize tumor antigens^22^. Therefore, CSC vaccines are extremely important for the treatment of T cell clones that do not recognize tumors. However, CAR-T adoptive cell immunotherapy has significant limitations. Although it has a good effect on leukemia and lymphoma, it has a poor effect on solid tumors. It can also induce toxic side effects such as cytokine release syndrome, neurological toxicity, anaphylaxis, and on-target/off-tumor recognition^23^. CSCs are important factors for completely curing tumors. At present, there is a lack of immune vaccines targeting these cells. The control of infectious diseases may be achieved by the development and application of various specific immune vaccines. The identification of specific markers for CSCs has been a research focus to enable the preparation of immune vaccines to target and kill CSCs through eliciting an immune response, thereby preventing the occurrence or recurrence of cancer. But the challenge remains that tumor cells are different from foreign microorganisms in that their specific protein immunogenicity is weak, the mechanism of tumor immune evasion is complex, and tumor cells are not easily recognized by the immune system. A promising approach is therefore to activate an immune response by coupling appropriate immune adjuvants with tumor-specific peptide epitopes.

The use of tumor stem cell-associated antigen epitope peptides as vaccines is a focus of the current research and has several advantages, including safety and efficacy in generating immune responses.

The OCT4 transcription factor plays a role in embryonic development and is necessary for embryonic stem cell pluripotency. OCT4 is deemed to be a good candidate TAA with many advantageous characteristics. First, OCT4 has been reported to be highly expressed in many tumors, such as carcinomas of the breast^24^, testis^25^, bladder^26^, germ-cell tumors^27^, and in CSCs^28^, but it is hardly expressed in the final, differentiated, normal tissues. Second, the overexpression of OCT4 is positively associated with poor prognosis and a shortened survival time of cancer patients^29^.

## Conclusions

Here we reported for the first time, a cancer vaccine based on a TAA, administered to test the immunogenicity and safety of OCT4 vaccines. In our study, KLH was used as a carrier and adjuvant system for the development of three different vaccines comprising the tumor-associated antigen epitope peptide OCT4. The vaccines were combined with TLR9 to enhance the immune response. *In vivo* studies demonstrated that OCT4-3 + TLR9 had the strongest antitumor activity and long-term survival advantage against OCT4^+^ F9 tumors. Additionally, OCT4-3 + TLR9 vaccine induced a strong, antigen-specific, CTL response against F9 cells. Importantly, the development of anti-OCT4 antibodies was not associated with significant adverse events.

## Figures and Tables

**Table 1:** Serum protein values after treatments

Sample	WBC (10^3^/mL)	RBC (%)	HGB (fL)	HCT (10^6^/mL)	MCV (g/dL)	PLT (10^3^/mL)
PBS	6.9 ± 1.5	11.1 ± 1.6	182.4 ± 21.9	56.7 ± 7.9	51.3 ± 0.8	574.4 ± 140.5
TLR9	6.1 ± 1.5	10.3 ± 1.0	166.8 ± 16.7	51.4 ± 5.3	49.9 ± 0.5	536.9 ± 158.9
OCT4-3	8.9 ± 2.5	10.3 ± 0.4	169.5 ± 6.6	52.6 ± 2.4	50.9 ± 0.4	597.3 ± 119.4
OCT4-3+TLR9	5.6 ± 1.4	9.9 ± 0.9	161 ± 13.1	52.2 ± 4.7	52.5 ± 0.9	773.7 ± 133.2

HCT, red blood cell specific volume; HGB, hemoglobin; MCV, mean corpuscular volume; PLT, platelet; RBC, red blood cells; TLR9, Toll-like receptor-9; WBC, white blood cells.

**Table 2:** Whole blood analysis after treatments

Sample	ALT (IU/L)	AST (IU/L)	ALP (IU/L)	BUN (mmol/L)	ALB (g/L)	GLU (mmol/L)	TBIL (mmol/L)	CREA (mmol/L)	TP (g/L)
PBS	36.0 ± 10.7	178.5 ± 55.4	127.8 ± 32.9	6.8 ± 1.7	30.3 ± 3.8	0.25 ± 0.2	15.4 ± 3.6	105 ± 25.4	62.8 ± 6.9
TLR9	42.0 ± 4.4	228.3 ± 57.5	171.1 ± 48.8	7.5 ± 1.5	35.6 ± 4.1	0.63 ± 0.1	16.9 ± 1.4	126.2 ± 10.7	66.8 ± 6.9
OCT4-3	42.9 ± 5.7	214.4 ± 66.1	145.7 ± 32.5	6.5 ± 1.1	36. 8 ± 1.8	0.13 ± 0.1	15.6 ± 1.1	121 ± 13.5	69.5 ± 2.1
OCT4-3+TLR9	41.6 ± 6.9	206.4 ± 43.6	150.3 ± 35.2	6.6 ± 0.8	33.5 ± 2.1	0.31 ± 0.0	18.2 ± 3.9	101.3 ± 36.4	69.3 ± 4.1

ALB, albumin; ALP, alkaline phosphatase; ALT, alanine aminotransferase; AST, aspartate aminotransferase; BUN, blood urea nitrogen; CREA, creatinine; GLU, glucose; TOLR9, Toll-like receptor-9; TBIL, total bilirubin levels; TP, total protein.

## References

[1] Bray F, Ferlay J, Soerjomataram I, Siegel RL, Torre LA, Jemal A (2018). Global cancer statistics 2018: GLOBOCAN estimates of incidence and mortality worldwide for 36 cancers in 185 countries.. Cancer J Clin..

[2] Sau S, Alsaab HO, Kashaw SK, Tatiparti K, Iyer AK (2017). Advances in antibody-drug conjugates: A new era of targeted cancer therapy.. Drug Discov Today..

[3] Yang J, Hu L (2019). Immunomodulators targeting the pd-1/pd-l1 protein–protein interaction: From antibodies to small molecules.. Med Res Rev..

[4] Blank CU, Enk A (2015). Therapeutic use of anti-CTLA-4 antibodies.. Int Immunol..

[5] Ikeda H (2016). T-cell adoptive immunotherapy using tumor-infiltrating T cells and genetically engineered TCR-T cells.. Int Immunol..

[6] Shoemaker RH, Forsthuber TG (2017). Targeting “retired antigens” for cancer immunoprevention.. Cancer Prev Res..

[7] Xiao YF, Jie MM, Li BS, Hu CJ, Xie R, Tang B (2015). Peptide-based treatment: A promising cancer therapy.. J Immunol Res..

[8] Ilyas S, Yang JC (2015). Landscape of tumor antigens in T cell immunotherapy.. J Immunol..

[9] Fridman WH, Pages F, Sautes-Fridman C, Galon J (2012). The immune contexture in human tumours: Impact on clinical outcome.. Nature Rev Cancer.

[10] Zhao Y, Feng F, Zhou YN (2015). Stem cells in gastric cancer.. World J Gastroenterol..

[11] Ge YJ, Fuchs E (2018). Stretching the limits: From homeostasis to stem cell plasticity in wound healing and cancer.. Nature Rev Genetics..

[12] Kim JB, Sebastiano V, Wu GM, Arauzo-Bravo MJ, Sasse P, Gentile L (2009). OCT4-induced pluripotency in adult neural stem cells.. Cell..

[13] Sterneckert J, Hoing S, Scholer HR (2012). Concise review: OCT4 and more: The reprogramming expressway.. Stem Cells..

[14] Kar S, Patra SK (2018). Overexpression of OCT4 induced by modulation of histone marks plays crucial role in breast cancer progression.. Gene..

[15] Cheng L, Sung MT, Cossu-Rocca P, Jones TD, MacLennan GT, De Jong J (2007). OCT4: Biological functions and clinical applications as a marker of germ cell neoplasia.. J Pathol..

[16] Zhao Y, Li CG, Huang L, Niu S, Lu QJ, Gong DJ (2018). Prognostic value of association of OCT4 with LEF1 expression in esophageal squamous cell carcinoma and their impact on epithelial-mesenchymal transition, invasion, and migration.. Cancer Med-US..

[17] Lin GM, Wang XM, Yi WX, Zhang CX, Xu GX, Zhu XM (2015). A conjugate of octamer-binding transcription factor 4 and toll-like receptor 7 agonist prevents the growth and metastasis of testis embryonic carcinoma.. J Transl Med..

[18] Ho HH, Li YH, Lee JC, Wang CW, Yu YL, Hueng DY (2018). Vestibular schwannomas: Accuracy of tumor volume estimated by ice cream cone formula using thin-sliced MR images.. Plos One..

[19] Wu Q, Chen X, Wang P, Wu Q, Qi X, Han X (2020). Delivery of arsenic trioxide by multifunction nanoparticles to improve the treatment of hepatocellular carcinoma.. ACS Appl Mater Interfaces..

[20] Chen TT, Hu YL, Liu B, Huang XP, Gao NN, Jin ZC (2016). Synthetic Toll-like receptor 7 agonist as a conjugated adjuvant enhances the TH1 type immunogenicity of influenza virus vaccine.. Int J Clin Exp PathoL..

[21] Cherepanova OA, Gomez D, Shankman LS, Swiatlowska P, Williams J, Sarmento OF (2016). Activation of the pluripotency factor OCT4 in smooth muscle cells is atheroprotective.. Nat Med.

[22] Tumeh PC, Harview CL, Yearley JH, Shintaku IP, Taylor EJM, Robert L (2014). Pd-1 blockade induces responses by inhibiting adaptive immune resistance.. Nature..

[23] Neelapu SS, Tummala S, Kebriaei P, Wierda W, Gutierrez C, Locke FL (2018). Chimeric antigen receptor T-cell therapy – assessment and management of toxicities.. Nature Rev Clin Oncol..

[24] Gwak JM, Kim M, Kim HJ, Jang MH, Park SY (2017). Expression of embryonal stem cell transcription factors in breast cancer: OCT4 as an indicator for poor clinical outcome and tamoxifen resistance.. Oncotarget..

[25] Roth LM, Michal M, Michal M, Cheng L (2018). Protein expression of the transcription factors dmrt1, tclf5, and OCT4 in selected germ cell neoplasms of the testis.. Hum Pathol..

[26] Huang P, Chen J, Wang L, Na YQ, Kaku H, Ueki H (2012). Implications of transcriptional factor, OCT-4, in human bladder malignancy and tumor recurrence.. Med Oncol..

[27] Cheng L (2004). Establishing a germ cell origin for metastatic tumors using OCT4 immunohistochemistry.. Cancer..

[28] Wang D, Lu P, Zhang H, Luo MN, Zhang X, Wei XF (2014). OCT-4 and NANOG promote the epithelial-mesenchymal transition of breast cancer stem cells and are associated with poor prognosis in breast cancer patients.. Oncotarget..

[29] Lee JH, Yun CW, Han YS, Kim S, Jeong D, Kwon HY (2018). Melatonin and 5-fluorouracil co-suppress colon cancer stem cells by regulating cellular prion protein-Oct4 axis. J Pineal Res..

